# Expression of PIK3CA, PTEN mRNA and *PIK3CA* mutations in primary breast cancer: association with lymph node metastases

**DOI:** 10.1186/2193-1801-2-464

**Published:** 2013-09-16

**Authors:** Irina Palimaru, Anja Brügmann, Marie Kim Wium-Andersen, Ebba Nexo, Boe Sandahl Sorensen

**Affiliations:** Department of Clinical Biochemistry, Aarhus University Hospital, Norrebrogade 44, Aarhus, 8000 Denmark; Department of Radiology, Skovlyvej 1, Regional Hospital of Randers, Randers, 8930 Denmark; Institute of Pathology, Aalborg University Hospital, Ladegaardsgade 3, Aalborg, 9100 Denmark; Department of Clinical Biochemistry, Herlev Hospital, Herlev Ringvej 75, Herlev, 2730 Denmark

**Keywords:** PI3K pathway, PIK3CA, Mutations, PTEN, Breast cancer, Lymph node metastases

## Abstract

**Purpose:**

High activity of the intracellular phosphatidylinositol-3 kinase (PI3K) pathway is common in breast cancer. Here, we explore differences in expression of important PI3K pathway regulators: the activator, phosphatidylinositol-3-kinase catalytic subunit alpha (PIK3CA), and the tumour suppressor, phosphatase and tensin homolog (PTEN), in breast carcinoma tissue and normal breast tissue. Furthermore, we examine whether expression of PIK3CA and PTEN mRNA and occurrence of *PIK3CA* mutations are associated with lymph node metastases in patients with primary breast cancer.

**Methods:**

Paired tissue samples of breast carcinoma and normal breast tissue were obtained from 175 breast cancer patients at the time of primary surgery, of these 105 patients were lymph node positive. Expression of PIK3CA and PTEN mRNA was quantified with Quantitative Real Time PCR. Somatic mutations in exon 9 and exon 20 of the *PIK3CA* gene were identified by genotyping.

**Results:**

Both PIK3CA and PTEN mRNA expression was significantly increased in breast carcinoma tissue compared to normal breast tissue (*p* = 2 × 10^-11^) and (*p* < 0.001), respectively. *PIK3CA* mutations were present in 68 out of 175 patients (39%), but were not associated with PIK3CA expression (*p* = 0.59). Expression of PIK3CA and PTEN mRNA, and *PIK3CA* mutations in breast carcinomas were not associated with presence of lymph node metastases.

**Conclusions:**

The expression of PTEN and PIK3CA mRNA is increased in breast carcinoma tissue compared to normal breast tissue, and *PIK3CA* mutations are frequent in primary breast carcinoma, however these factors were not associated with lymph node metastases.

**Electronic supplementary material:**

The online version of this article (doi:10.1186/2193-1801-2-464) contains supplementary material, which is available to authorized users.

## Background

The phosphatidylinositol-3 kinase (PI3K) intracellular pathway promotes cell survival, proliferation and growth (Engelman et al. 2006). It is one of the most commonly altered pathways in human cancers (Samuels et al. 2004; Cully et al. 2006) and plays a major role in breast cancer development and progression.(Campbell et al. 2004; Bachman et al. 2004; McAuliffe et al. 2010; Boyault et al. 2012).

The PI3K is composed of two subunits: An 85 kDa regulatory subunit and an 110 kDa catalytic subunit. The catalytic subunit PIK3CA is encoded by the *PIK3CA* gene, and is the main regulator of PI3K activation. Activation of PI3K leads to downstream signalling through a series of serine/threonine kinases, resulting in increased cell survival and proliferation (Engelman et al. 2006). Another key regulator of the PI3K pathway is the lipid phosphatase and tensin homolog (PTEN) encoded by the *PTEN* gene. PTEN inhibits activation of the PI3K protein (Cully et al. 2006).

Somatic mutations in the *PIK3CA* gene often result in increased activity of PIK3CA, and the gene is thus regarded as a transforming oncogene (Boyault et al. 2012). *PIK3CA* mutations are reported in 18%–40% of breast cancers (Kalinsky et al. 2009; Cizkova et al. 2012; Harle et al. 2013), most frequently in exon 9 and 20 (Campbell et al. 2004; Samuels et al. 2004).

The association between *PIK3CA* mutations and lymph node metastases is still unclear. Saal et al. reported an association between *PIK3CA* mutations and lymph node metastases (Saal et al. 2005), and this was supported by a large study which reported an association between expression of the PIK3CA and lymph node metastases (Aleskandarany et al. 2010). However, results are conflicting as other studies have failed to find such associations (Buttitta et al. 2006; Maruyama et al. 2007; Cizkova et al. 2012). In addition, the study by Kalinsky et al. (2009) showed an association between *PIK3CA* mutations and absence of lymph node metastases.

In this study, we examine the mRNA expression of the key regulators of the PI3K signalling pathway, PIK3CA, PTEN, and the occurrence of *PIK3CA* mutations in breast cancer and corresponding normal tissue from patients with primary breast cancer, and we relate the results to the presence of lymph node metastases.

## Materials and methods

### Study population and samples

This study from 2008–2009 includes 175 women with primary breast cancer. Of these, 105 were lymph node positive. Inclusion criteria were age >18 years and an operable primary breast cancer. Patients previously treated with neoadjuvant therapy were excluded. Permission from the Danish Research Ethics Committee (N-20070047) and the Danish Data Protection Agency (2011-41-6930) was obtained in 2007 and 2011 respectively. All patients signed informed consent. The inclusion of patients took place during the first round of breast cancer screening in Denmark and consequently the percentage of patients with lymph node metastases is higher than expected.

**Figure 1:**
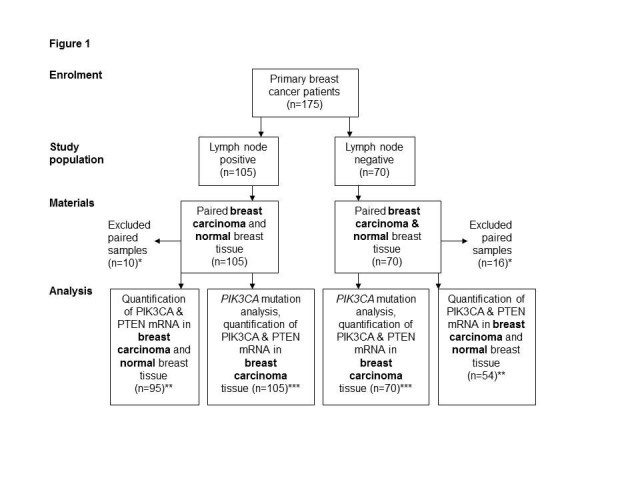
**Flowchart illustrating the materials and the analyses performed.** * Exclusion was due to insufficient amount or quality of RNA. ** Data are shown in Figures 2 and 3. *** Data are shown in Tables 1, 2 and 3.

Tissue samples from the breast carcinoma and the normal breast tissue were obtained from each patient at the time of primary surgery. Due to insufficient amount or quality of RNA, 26 patients were excluded from the paired analyses (Figure 1), which therefore included only 149 patients. All tissue samples were immediately frozen and stored at −80°C.

### Extraction of mRNA and preparation of cDNA

Total RNA was extracted from all tissue samples employing the total RNA kit from Qiagen^©^, (Qiagen, Hilden, Germany, http://www.qiagen.com) according to the manufacturer’s instructions.

cDNA was synthesized from 1 μg total RNA in a 20 μl reaction mix including 50 μmol/l Oligo(dT), reverse transcriptase (50 units/μl), RNase inhibitors (20 units/μl), 0.4 mmol/l of each dNTP, 1 × PCR buffer, and 25 mmol/l MgCL_2_. All reagents were from Applied Biosystems® (Applied Biosystems Inc., CA, USA). Reverse transcription was performed on the Perkin-Elmer GeneAmp® PCR System 9600 Thermal Cycler (PerkinElmer Inc., MA, USA) with the profile: 42°C for 30 minutes, 99°C for 5 minutes and 4°C until samples had cooled. cDNA was stored at −20°C until further use.

### Quantification of PIK3CA, PTEN and HMBS mRNA

Quantitative Real Time PCR (qPCR) was used to quantify PIK3CA, PTEN and hydroxy-methyl-bilane synthase (HMBS) cDNA with the LightCycler® 480 (LC480) Real-Time PCR System from Roche (Roche Applied Science, Mannheim, Germany, http://www.roche-applied-science.com). HMBS was used to control for variations in RNA concentration and integrity and was found to be the best suited household gene when compared to β-Actin (ACTB), glyceraldehyde-3-phosphate dehydrogenase (GAPDH), tyrosine 3-monooxygenase/tryptophan 5-monooxygenase activation protein zeta polypeptide (YWHAZ) and beta-2-microglobulin (B2M) according to the Normfinder method (Andersen et al., 2004). The reaction mix consisted of 5 μl SYBR® Green I Master Mix Buffer (SYBR® Green RT-PCR Reagents Kit, Roche, Mannheim, Germany), 0.5 pmol forward and reverse primers (Eurofins MWG Synthesis GmbH, Ebersberg, Germany), 1 μl cDNA and H_2_0 to a final volume of 10 μl. The primers were designed to let the PCR product span intron sequences in order to avoid DNA contamination. Primer sequences and accession numbers are listed in Additional file 1: Table S1. qPCR was performed with the following profile: 95°C for 10 min and a total number of 50 cycles with an annealing temperature of 66°C when using PIK3CA primers, 64°C when using PTEN primers and 59°C when using the HMBS primers. However all products were detected before cycle 32. Following amplification, a melting curve was obtained to provide evidence for a single reaction product. The Roche® LC480 Software Version 1.5.0.39 (Roche Applied Science, Mannheim Germany) was used for quantification and melting temperature determination. A no-template control (nuclease-free water instead of RNA) was included in each run. Serial dilutions of RNA from the breast cancer cell line MDA MB (from ATCC) were used as calibrators.

### Analysis of *PIK3CA* mutations

We genotyped the *PIK3CA* gene at exon 9 and exon 20 where > 90% of all *PIK3CA* mutations are clustered (Campbell et al. 2004; Samuels et al. 2004). We used the PI3K Mutation Test Kit from Qiagen© (Qiagen, Hilden, Germany, http://www.qiagen.com), which contained one control assay and three mutations assays to be run in a qPCR assay. The qPCR was performed on cDNA from all breast carcinoma samples using the Roche® LC 480 instrument.

### Pathological examination

All tissue samples were examined by specialists in breast pathology at the Institute of Pathology, Aalborg University Hospital. Tumour size and histological type (invasive ductal; invasive lobular; other types) were evaluated according to the Danish Breast Cancer (DBCG) guidelines. Oestrogen receptor (ER) status was determined by immunohistochemistry (IHC). Human epidermal growth factor receptor 2 (HER2) status was determined by IHC and fluorescence in situ hybridization (FISH).

### Statistical analysis

We used a paired *t*-test of log-transformed concentrations of PIK3CA and PTEN mRNA to compare expression in breast carcinoma tissue and normal breast tissue. To test for associations between *PIK3CA* mutations and/or expression of PIK3CA and/or PTEN and lymph node metastases, we used four different approaches: First, we log-transformed PIK3CA and PTEN levels and used an unpaired *t*-test to compare expression of PIK3CA and PTEN in breast carcinoma tissue from patients with and without lymph node metastases and to compare levels of PIK3CA expression in patients with and without *PIK3CA* mutations. Second, patients were divided in two groups using the median value of either PIK3CA or PTEN as cut-off. We used a chi^2^ test to test whether elevated expression of PIK3CA or PTEN in breast carcinoma associated with lymph node metastases. We repeated analyses with patients stratified according to *PIK3CA* mutations in breast carcinoma tissue (one or more mutations vs. no mutations, *“* wild-type*”*). Third, patients were stratified into groups according to their number of risk factors. Risk factors were defined as PIK3CA expression above median, PTEN expression below median, and *PIK3CA* mutations. We used a Cuzick’s extension of the Wilcoxon rank sum test to calculate *p*-trend across groups. Finally, we calculated risk of lymph node metastases based on the binary variables of PIK3CA and PTEN expression and *PIK3CA* mutations using two different logistic regression models to calculate odds ratios (ORs) with 95% confidence intervals. The models were 1) unadjusted and 2) adjusted for age at surgery, tumour size, tumour histology, ER status, and HER2 status.

Stata 12.0 (StataCorp LP, College Station, TX, USA) was used for all statistical analyses and GraphPad Prism 4.03 (GraphPad Software Inc., CA, USA) was used for depicting the results.

## Results

The clinical and pathological characteristics of all 175 patients are listed in Table 1.

**Table 1:** **Clinical pathological characteristics of the 175 study participants in relation to levels of PIK3CA-, PTEN mRNA expression and** ***PIK3CA*** **mutations in the primary breast carcinoma**

	PIK3CA expression (n = 175)		PTEN expression (n = 175)		PIK3CA mutations (n = 175)	
	Low PIK3CA	High PIK3CA	Low PTEN	High PTEN	Wildtype	Mutations
**Number, N (%)**	87 (50)	88 (50)	87 (50)	88 (50)	107 (61)	68 (39)
**Age, years** median (range)	64 (38–85)	65 (32–83)	64 (36–85)	63 (32–82)	64 (32–85)	63 (38–83)
**Tumour size,** mm, median (range)	21 (6–60)	18 (6–51)	21 (6–60)	18 (6–50)	19 (6–55)	19 (7–60)
**Oestrogen receptor status, N (%)**						
Positive	74 (85)	79 (90)	74 (85)	79 (90)	92 (86)	61 (90)
Negative	13 (15)	9 (10)	13 (15)	9 (10)	15 (14)	7 (10)
**HER2 status, N (%)**						
Normal expression	65 (74)	69 (78)	59 (68)	75 (85)	81 (76)	53 (78)
Overexpression	10 (11)	11 (13)	11 (13)	10 (11)	13 (12)	8 (12)
Unknown	12 (14)	8 (9)	17 (19)	3 (3)	13 (12)	7 (10)
**Histology, N (%)**						
Invasive ductal carcinoma	68 (78)	70 (80)	71 (82)	67 (76)	88 (82)	50 (74)
Invasive lobular carcinoma	10 (11)	15 (17)	8 (9)	17 (19)	14 (13)	11 (16)
Other	9 (10)	3 (3)	8 (9)	4 (5)	5 (5)	7 (10)

Low PIK3CA expression is PIK3CA expression ≤ 50 (median) high expressions is values above 50. Low PTEN expression is PTEN expression ≤ 23. (median). High values are values above 23 Wildtype = no *PIK3CA* mutations. *HER2* human epidermal growth factor receptor 2.HER2 overexpression is score 2+ and 3+ after FISH amplification.

### PIK3CA and PTEN mRNA expression in breast carcinoma and in normal breast tissue in 149 patients

**Figure 2:**
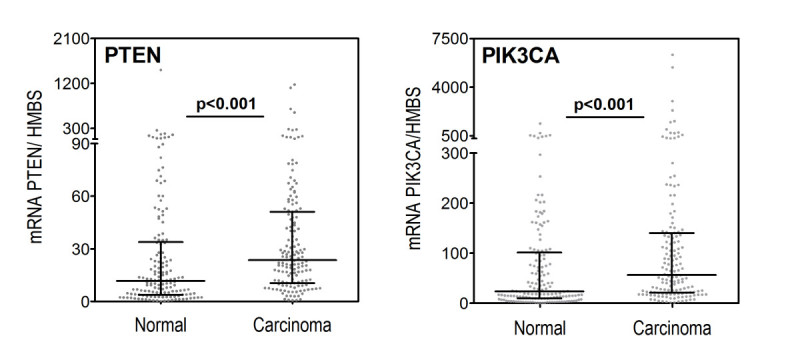
**PIK3CA and PTEN mRNA expression in paired normal and breast carcinoma tissue from 149 patients (n = 95 lymph node positive and n = 54 lymph node negative).**

PIK3CA mRNA expression in breast carcinoma tissue was significantly higher than in normal breast tissue (p = 2 × 10^-11^), with a median value of 56 for the PIK3CA/HMBS ratio in breast carcinoma tissue compared to 23 PIK3CA/HMBS in normal breast tissue (Figure 2) PIK3CA mRNA expression was increased in tumour tissue in 114 out of 149 patients (76%).

Unexpectedly, PTEN mRNA expression in breast carcinoma tissue was significantly higher than in normal breast tissue (p = 2 × 10^-11^), with a median value of 24 for the PTEN/HMBS ratio in breast carcinoma tissue compared to 12 PTEN/HMBS in the normal breast tissue. PTEN mRNA expression was reduced in tumour tissue in only 31 out of 149 patients (20%). When we examined PIK3CA and PTEN mRNA expression in different strata of the study population: By oestrogen receptor status, HER2 status, and by tumour histology, results were similar (Additional file 1: Table S2).

### PIK3CA, PTEN mRNA expression and *PIK3CA* mutations in relation to lymph node metastases

**Figure 3:**
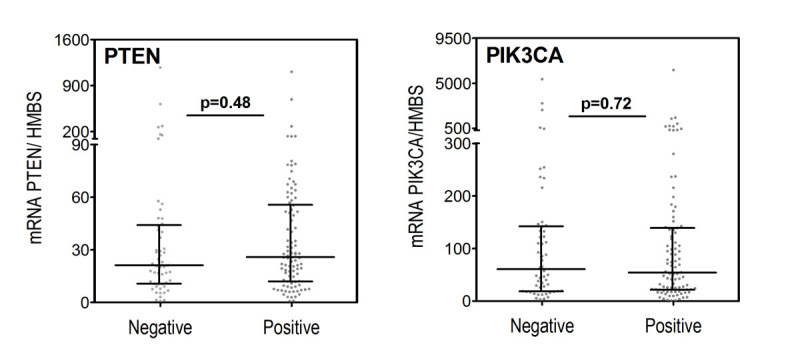
**PIK3CA and PTEN mRNA expression in breast carcinoma tissue from 149 patients in relation to lymph node metastases, (n = 95 lymph node positive and n = 54 lymph node negative).**

Levels of PIK3CA and PTEN expression in breast carcinoma tissue were similar in patients with and without lymph node metastases (p = 0.72 and p = 0.48) (Figure 3) There was no association between elevated PIK3CA expression and lymph node metastases (p = 0.71) or between reduced PTEN expression and lymph node metastases (p = 0.32) (Table 2).

**Table 2:** **Lymph-node status of the 175 study participants in relation to levels of PIK3CA-, PTEN mRNA expression and** ***PIK3CA*** **mutations in the primary breast carcinoma**

	PIK3CA expression		PTEN expression		PIK3CA mutations	
	Low PIK3CA	High PIK3CA	Low PTEN	High PTEN	Wildtype	Mutations
**Number, N (%)**	87 (50)	88 (50)	87 (50)	88 (50)	107 (61)	68 (39)
**Lymph node status, N (%)**						
No metastases	36 (41)	34 (39)	38 (44)	32 (36)	42 (39)	28 (41)
Metastases	51 (59)	54 (61)	49 (56)	56 (64)	65 (61)	40 (59)
	p = 0.71		p = 0.32		p = 0.80	

Low PIK3CA expression is PIK3CA expression ≤ 50.60 (median). Low PTEN expression is PTEN expression ≤ 22.98 (median).

Wildtype = no *PIK3CA* mutations. *HER2* human epidermal growth factor receptor 2.

Mutations in the *PIK3CA* gene were present in 68 (39%) of 175 patients. The most frequent location was in exon 20 located at H1074R (54%) and less frequent in exon 9 at E545K/D (31%) and E542K (15%). Patients carrying *PIK3CA* mutations had higher PIK3CA expression (median of 62 vs. 47), but this was not significant (p = 0.59) (Additional file 1: Table S3). There was no association between *PIK3CA* mutations and lymph node metastases (p = 0.80) (Table 2).

We stratified the patients according to number of risk factors (PIK3CA expression above median, PTEN expression below median, and presence of *PIK3CA* mutations) and found that 13 (7%) had no risk factors, 91 (52%) had one risk factor, 61 (35%) had two risk factors, and 10 (6%) had three risk factors. We did not find an increased risk of lymph node metastases across groups (p-trend = 0.543) (Table 3).

**Table 3:** **Lymph-node status of the 175 study participants with primary breast cancer in relation to one or more potential risk factors**

	No. of potential risk factors					
	0	1	2	3	Total	p-trend
**Number, N (%)**	13 (7)	91 (52)	61 (35)	10 (6)	175 (100)	
**Lymph node status, N (%)**						
No metastases	5 (38)	36 (40)	23 (38)	6 (60)	70 (40)	0.543
Metastases	8 (62)	55 (60)	38 (62)	4 (40)	105 (60)	

Potential risk factors are: PIK3CA expression > 50.60 (median), PTEN expression ≤ 22.98 (median), and *PIK3CA* mutations.

Finally, the unadjusted odds ratio (OR) was 1.12 (95% confidence interval (CI) 0.61-2.05) for patients with PIK3CA expression above median level compared to patients with PIK3CA expression below or at this level. After multifactorial adjustment for age, tumour size, histology, ER status, and HER2 status, the OR was 1.36 (0.71-2.59) (Additional file 1: Table S4). The corresponding ORs were 0.73 (0.40-1.35) and 0.57 (0.29-1.11) for patients with PTEN expression below or at the median level compared to patients with PTEN expression above this level, and 0.92 (0.49-1.72) and 0.96 (0.50-1.82) for patients with *PIK3CA* mutations compared to patients without.

## Discussion

In this study, we examined important regulators of the PI3K pathway in primary breast carcinoma and normal breast tissue to test whether an increased expression of the PI3K pathway activator, PIK3CA, a reduced expression of the tumour suppressor, PTEN, and the presence of *PIK3CA* mutations in breast carcinoma tissue are associated with lymph node metastases.

While most breast cancer studies examined the role of the *PIK3CA* mutations, less attention has been given to PIK3CA expression. To our knowledge, no other study has examined the PIK3CA expression in both breast carcinoma tissue and corresponding normal breast tissue. Our data showed significantly higher PIK3CA expression in breast carcinoma tissue than in normal breast tissue, a consistent finding even after stratifying for oestrogen receptor and HER2 receptor status as well as for tumour histology. This finding supports the hypothesis of an activated PI3K pathway in cancer cells, and it further supports the analysis of mRNA expression for detecting intracellular protein levels as an addition to direct protein measurements using IHC. Further studies are needed to explore the direct relation between expression of PIK3CA on mRNA and protein levels.

We did, however, fail to find association between PIK3CA expression and lymph node metastases. Our results conflict with a study of 1394 patients with primary operable breast cancer. The authors used IHC for measurement of PIK3CA and reported that elevated PIK3CA expression associated with a poor prognosis including lymph node metastases (Aleskandarany et al. 2010). Further studies are needed in order to unravel this discrepancy.

Unexpectedly, we did not find a decreased PTEN mRNA expression in tumour tissue. Our data showed a low PTEN mRNA expression in breast carcinoma tissue compared to the expression in normal breast tissue in only 20% of our paired cohort of 149 patients. In a study of 85 primary breast cancer patients using IHC for the detection of PTEN, a reduced or absent PTEN expression in breast carcinoma tissue compared to normal tissue was found in only 33% of the patients (Engin et al. 2006). Five other studies also detected low PTEN expression in primary breast carcinomas with IHC (Perren et al. 1999; Depowski et al. 2001; Lin et al. 2003; Lee et al. 2004; Gonzalez-Angulo et al. 2011), but these studies did not examine PTEN expression in the corresponding normal breast tissue.

We did not find any association between reduced PTEN and positive lymph node status. Previous results have been conflicting. The study mentioned above with 85 patients reported no association (Engin et al. 2006), while two other studies found an association between reduced PTEN in tumour tissue and lymph node metastases (Depowski et al. 2001; Lee et al. 2004). However, compared to our study population, the patients in these studies had a larger mean tumour size (Lee et al. 2004) and a more advanced cancer (tumour stages III and IV) (Depowski et al. 2001).

Finally, we found that *PIK3CA* mutations are frequent in primary breast cancer but were not associated with lymph node metastases. Of our patients, 39% had *PIK3CA* mutations, which is similar to the prevalence of 18%–40% reported in previous studies (Campbell et al. 2004; Bachman et al. 2004;Saal et al. 2005; Cully et al. 2006; Boyault et al. 2012). In line with previous studies, most mutations were located in exon 20 (Bachman et al. 2004; Saal et al. 2005; Maruyama et al. 2007; Kalinsky et al. 2009) and not associated with lymph node metastases (Campbell et al. 2004; Buttitta et al. 2006; Maruyama et al. 2007; Kalinsky et al. 2009; Boyault et al. 2012; Cizkova et al. 2012). However, one study of 160 Swedish primary breast cancer patients reported an association between *PIK3CA* mutations and lymph node metastases. Compared to our study, the prevalence of human epidermal growth factor receptor 2 (HER2) positive patients was twice as high as in the Swedish study population.

An important strength of our study is the paired material, both breast carcinoma tissue and normal breast tissue. Second, all samples were collected at the time of surgery before the patients received other treatments (e.g. neoadjuvant therapy), which might influence the regulation and activity of the PI3K signalling pathway (Gonzalez-Angulo et al. 2011). Third, while most other studies used IHC (Perren et al. 1999; Depowski et al. 2001; Lin et al. 2003; Lee et al. 2004; Engin et al. 2006; Aleskandarany et al. 2010; Gonzalez-Angulo et al. 2011), we used qPCR to determine expression of PTEN and PIK3CA, which enables quantification relative to a household gene and is not biased by subjective factors such as the experience of the assessor (Tvrdik et al. 2012; Mendoza et al. 2013).

A potential limitation of our study is the limited number of patients. Furthermore, almost 15% of all paired tissue samples were unavailable due to insufficient amount or quality of RNA after our qPCR analysis. Because the loss of data was most likely completely at random, we do not expect it to bias our results. Another potential limitation is that we did not have sufficient amount of tissue to measure protein levels of PIK3CA and PTEN and therefore we used the less tissue consuming mRNA expression technique, assuming that mRNA expression corresponds to the protein levels.

## Conclusions

In conclusion, we found significantly increased PIK3CA expression in breast carcinoma tissue compared to normal breast tissue. Unexpectedly, we also found increased PTEN expression in breast carcinoma tissue. An overactive PI3K pathway caused by increased PIK3CA expression, reduced PTEN expression, and *PIK3CA* mutations were not found to be associated with lymph node metastases.

## Electronic supplementary material

Additional file 1: Table S1: The Gene-specific primers used for the quantification analysis. **Table S2.** The association between high PIK3CA mRNA expression & *PIK3CA* mutations in the breast carcinoma of the 175 study participants. **Table S3.** The risk of lymph node metastases and levels of PIK3CA-, PTEN mRNA expression and *PIK3CA* mutations in the breast carcinoma of the 175 study participants. (DOCX 23 KB)

Below are the links to the authors’ original submitted files for images.

Authors’ original file for figure 1

Authors’ original file for figure 2

Authors’ original file for figure 3
