# Supplementary material for: Expression of PIK3CA, PTEN mRNA and PIK3CA mutations in primary breast cancer: association with lymph node metastases
Source: Springerplus. 2013 Sep 16;2(1):464. doi: 10.1186/2193-1801-2-464 (PMC3786083; doi:10.1186/2193-1801-2-464)
Supplement: Supplementary file 1 — Additional file 1: Table S1: The Gene-specific primers used for the quantification analysis. Table S2. The association between high PIK3CA mRNA expression & PIK3CA mutations in the breast carcinoma of the 175 study participants. Table S3. The risk of lymph node metastases and levels of PIK3CA-, PTEN mRNA expression and PIK3CA mutations in the breast carcinoma of the 175 study participants. (DOCX 23 KB) [file 40064_2013_520_MOESM1_ESM.docx]

Expression of PIK3CA, PTEN mRNA and *PIK3CA* mutations in primary breast cancer: Association with lymph node metastases

**Irina Palimaru^1,2^, Anja Brügmann^3^, Marie Kim Wium-Andersen^4^, Ebba Nexo^1^, Boe Sandahl Sorensen^1^**

**Supplementary data**

**Supplementary Table 1-3**

**Supplementary Table 1:** The Gene-specific primers used for the quantification analysis

**Supplementary Table 2:** The association between high PIK3CA mRNA expression & *PIK3CA* mutations in the breast carcinoma of the 175 study participants

**Supplementary Table 3:** The risk of lymph node metastases and levels of PIK3CA-, PTEN mRNA expression and *PIK3CA* mutations in the breast carcinoma of the 175 study participants

**Supplementary Table 1**

| **Gene symbol** | **Accession number** | **Primer Sequence**  **(5´-3´)** | **Amplicon size (bp)** |
| --- | --- | --- | --- |
| ***PIK3CA** | NM_006218.2 | Forward:GGCCACTGTGGTTGAATTGGGA | 250 |
| ***PIK3CA** | NM_006218.2 | Reverse: AGTGCACCTTTCAAGCCGCC | 250 |
| ****PTEN** | NM_000314.4 | Forward:TGGGCCCTGTACCATCCCAAGT | 445 |
| ****PTEN** | NM_000314.4 | Reverse: TGTGGCAACCACAGCCATCGT | 445 |
| *****HMBS** | NM­_000190.3 | Forward:CGGTACCCACGCGAATCAC | 64 |
| *****HMBS** | NM­_000190.3 | Reverse:GGGTACCCACGCGAATCAC | 64 |
| *Phospoinositide-3-kinase, catalytic, alpha polypeptide | | |  |
| **Phosphatase and tensin homolog | | |  |
| ***Hydroxy-methyl-bilane synthase | | |  |

**Supplementary Table 2**

|  | ***PIK3CA* mutations** | |  |
| --- | --- | --- | --- |
|  | wildtype | mutations |  |
| **Number, N (%)** | 107 (61) | 68 (39) |  |
|  |  |  |  |
| **PIK3CA expression, median (IQR)** | 46.6 (18.4-122.5) | 61.5 (19.9-141.2) |  |
|  |  |  |  |
|  | p=0.590 | |  |
|  |  |  |  |
| Wildtype = no *PIK3CA* mutations | | |  |

**Supplementary Table 3.**

|  | **Risk of Lymph node metastases** | | |  |
| --- | --- | --- | --- | --- |
|  | Unadjusted | | Multifactorially adjusted | |
|  | OR (95% CI) | p-value | OR (95% CI) | p-value |
| **PIK3CA expression*** |  |  |  |  |
| ≤ 50.604 (median) | 1 [reference] |  | 1 [reference] |  |
| > 50.604 | 1.12 (0.61-2.05) | 0.71 | 1.36 (0.71-2.59) | 0.35 |
|  |  |  |  |  |
| **PTEN expression**** |  |  |  |  |
| > 22.978 (median) | 1 [reference] |  | 1 [reference] |  |
| ≤ 22.978 | 0.73 (0.40-1.35) | 0.32 | 0.57 (0.29-1.11) | 0.10 |
|  |  |  |  |  |
| ***PIK3CA* mutations** |  |  |  |  |
| Wildtype | 1 [reference] |  | 1 [reference] |  |
| Mutations | 0.92 (0.49-1.72) | 0.80 | 0.96 (0.50-1.82) | 0.85 |
|  |  |  |  |  |
| *PIK3CA expression/HBMS. **PTEN expression/HBMS.  Wildtype = no *PIK3CA* mutations. | | | | |
| Multifactorially adjusted for age, tumour size, oestrogen receptor status, HER2 status  and histology. | | | | |
| HER2 = human epidermal growth factor receptor 2. | |  |  |  |
