# Supplementary figures and images for: Expression of PIK3CA, PTEN mRNA and PIK3CA mutations in primary breast cancer: association with lymph node metastases
Source: Springerplus. 2013 Sep 16;2(1):464. doi: 10.1186/2193-1801-2-464 (PMC3786083; doi:10.1186/2193-1801-2-464)

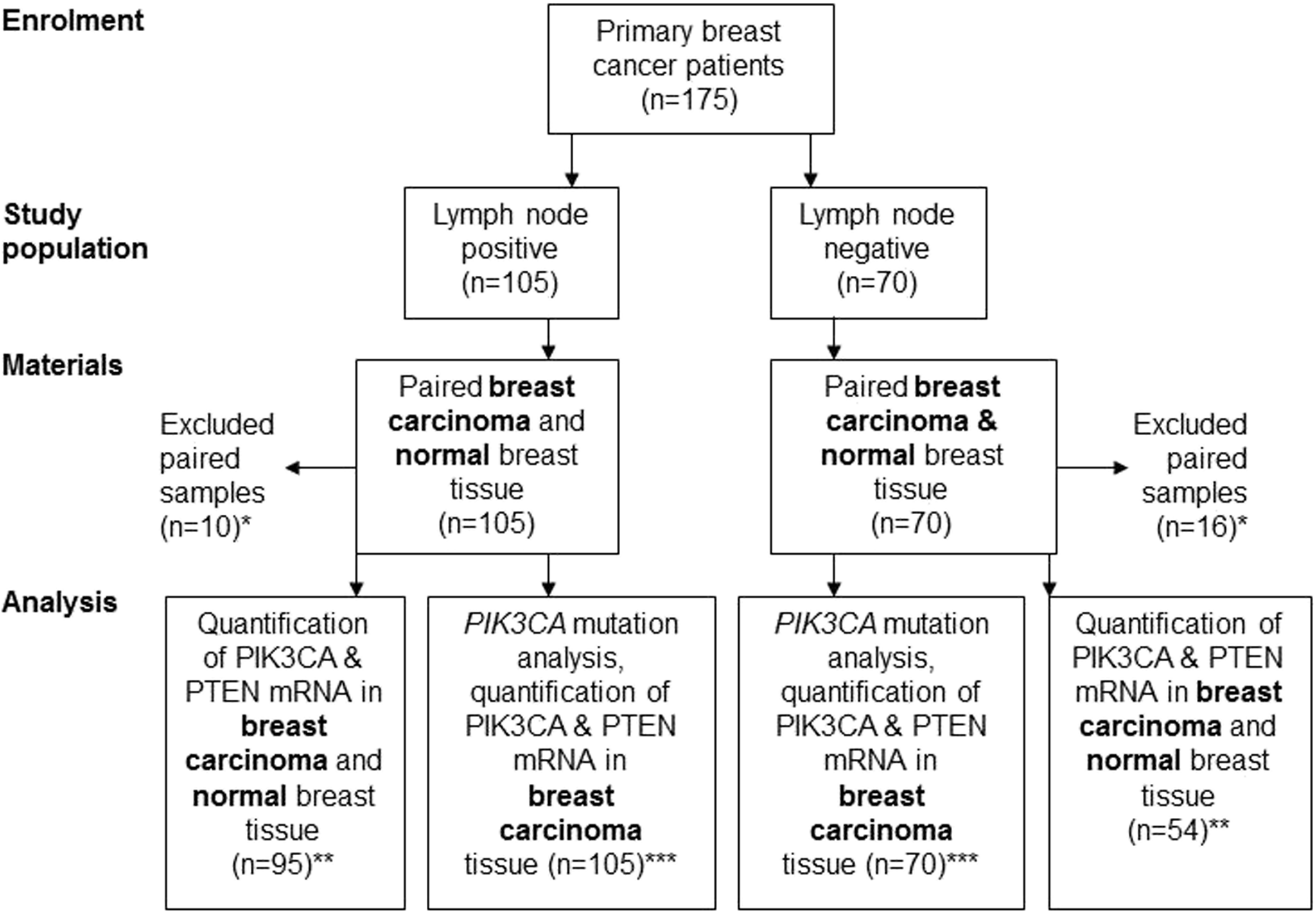

Supplement: Supplementary file 2 — Authors’ original file for figure 1 [file 40064_2013_520_MOESM2_ESM.tif]

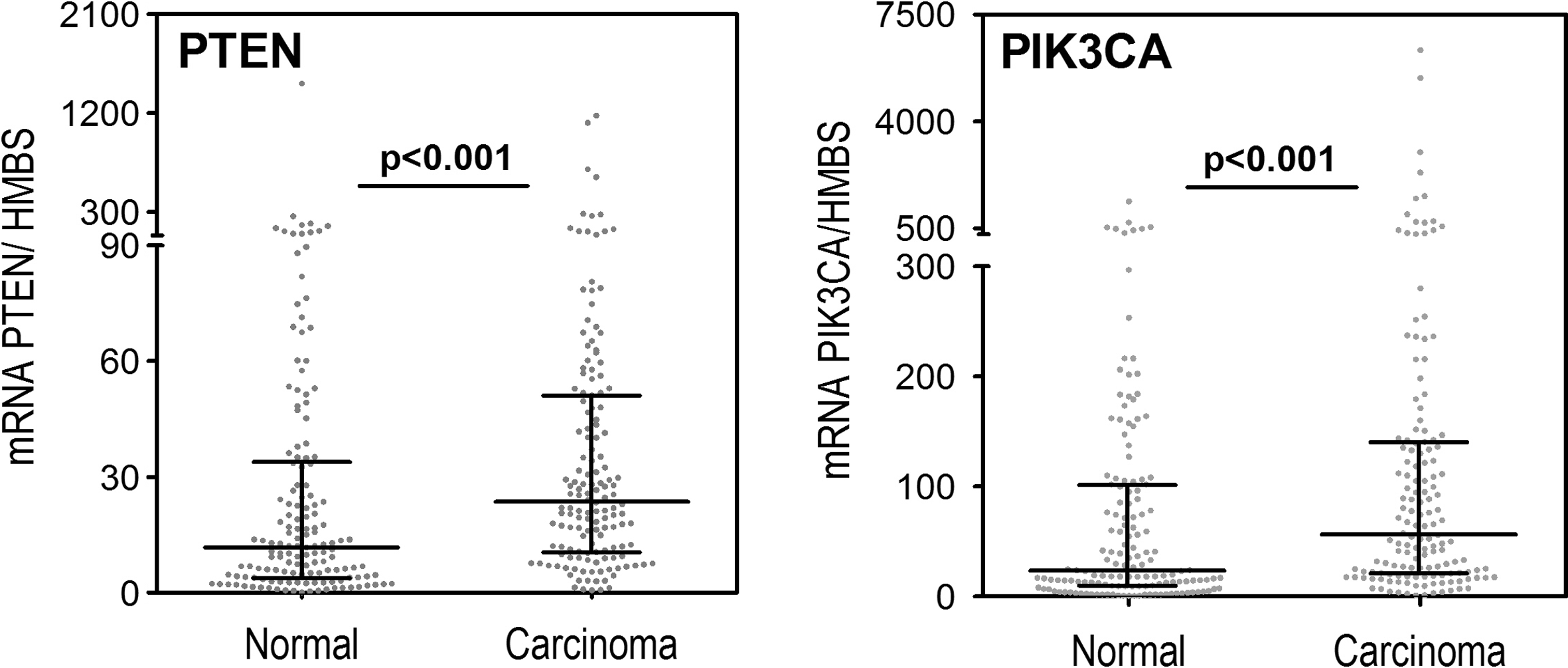

Supplement: Supplementary file 3 — Authors’ original file for figure 2 [file 40064_2013_520_MOESM3_ESM.tif]

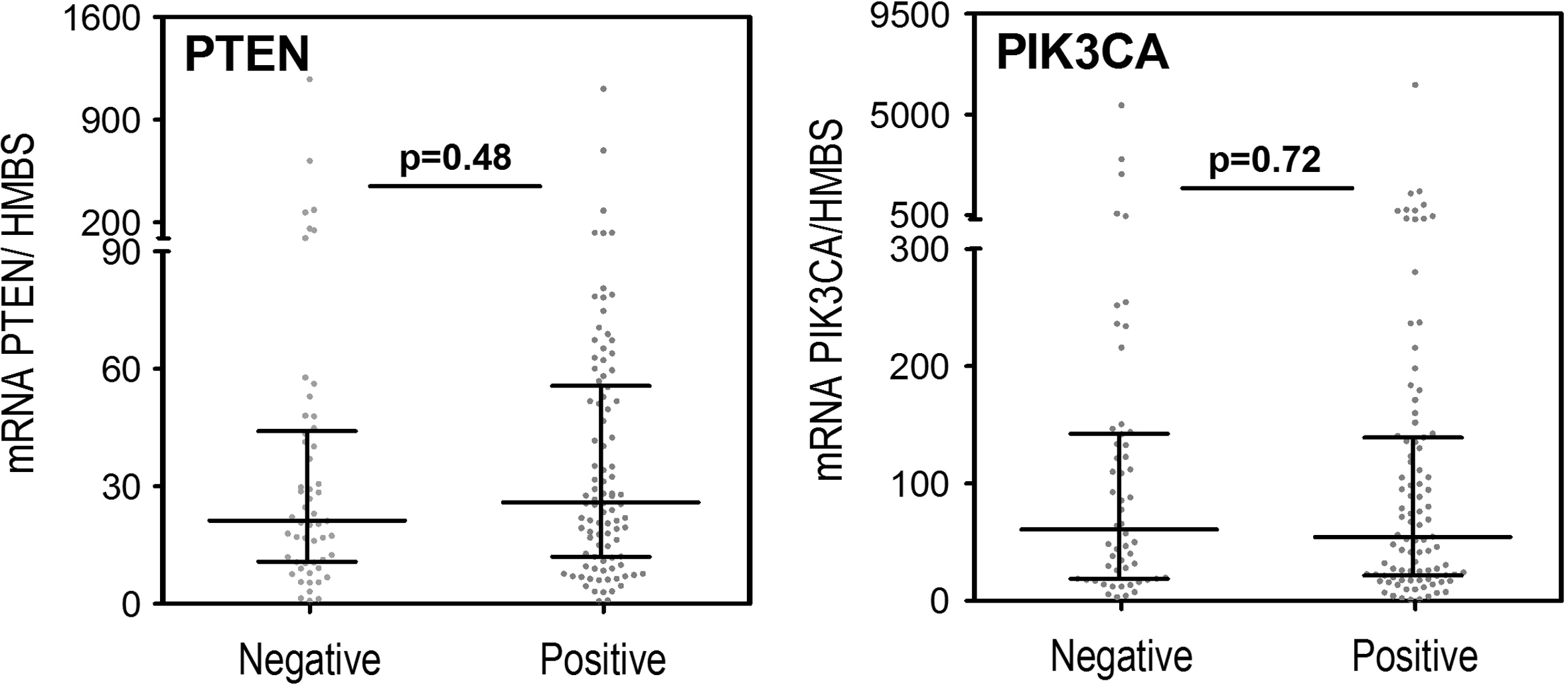

Supplement: Supplementary file 4 — Authors’ original file for figure 3 [file 40064_2013_520_MOESM4_ESM.tif]
